# Correction: Mn-ferrite nanoparticles as promising magnetic tags for radiofrequency inductive detection and quantification in lateral flow assays

**DOI:** 10.1039/d4na90118e

**Published:** 2024-11-25

**Authors:** Vanessa Pilati, María Salvador, Leyre Bei Fraile, José Luis Marqués-Fernández, Franciscarlos Gomes da Silva, Mona Fadel, Ricardo López Antón, María del Puerto Morales, José Carlos Martinez-García, Montserrat Rivas

**Affiliations:** a Departamento de Física, Universidad de Oviedo Campus de Viesques Gijón 33204 Spain pilativanessa@uniovi.es; b Complex Fluids Group, Instituto de Física & Faculdade UnB – Planaltina, Universidade de Brasília Brasília 70910-900 Brazil; c Departamento de Nanociencia y Nanotecnología, Instituto de Ciencia de Materiales de Madrid (ICMM) Madrid 28049 Spain; d Instituto Regional de Investigación Científica Aplicada (IRICA) and Departamento de Física Aplicada, Universidad de Castilla-La Mancha Ciudad Real Spain; e Instituto Universitario de Tecnología Industrial de Asturias (IUTA) Gijón 33203 Spain

## Abstract

Correction for ‘Mn-ferrite nanoparticles as promising magnetic tags for radiofrequency inductive detection and quantification in lateral flow assays’ by Vanessa Pilati *et al.*, *Nanoscale Adv.*, 2024, **6**, 4247–4258, https://doi.org/10.1039/D4NA00445K.

The authors regret that eqn (2) was incorrectly shown as a duplication of eqn (3). The correct eqn (2) is as follows:


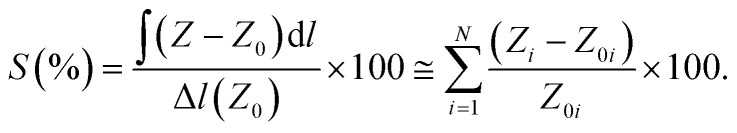
(2)

Eqn (3) remains correct and therefore unchanged.

Additionally references to the sensor resolution within the section named ‘3.4. Inductive response of the NPs in the sensor’ had been incorrectly expressed as nanograms (ng), whereas they should be micrograms (μg). The corrected text can be seen below.

‘We obtained a resolution of only 0.72 μg for S2 NPs and 0.87 μg for S1 NPs, outperforming our previous best result of 1.6 μg for magnetite nanoclusters.^7^’

The Royal Society of Chemistry apologises for these errors and any consequent inconvenience to authors and readers.

